# Healthcare Voucher Scheme for Screening of Cardiovascular Risk Factors: A Population-Based Study

**DOI:** 10.3390/ijerph182010844

**Published:** 2021-10-15

**Authors:** Junjie Huang, Chun-Ho Ngai, Man-Sing Tin, Qingjie Sun, Pamela Tin, Eng-Kiong Yeoh, Martin C. S. Wong

**Affiliations:** 1 The Jockey Club School of Public Health and Primary Care, Faculty of Medicine, Chinese University of Hong Kong, Hong Kong SAR 999077, China; junjie_huang@link.cuhk.edu.hk (J.H.); alfonse.ngai@gmail.com (C.-H.N.); mstin0911@gmail.com (M.-S.T.); stellarsun@cuhk.edu.hk (Q.S.); 2 Department of Healthcare and Social Development Research, Our Hong Kong Foundation, Hong Kong SAR 999077, China; pamela.tin@ourhkfoundation.org.hk; 3 School of Public Health, The Chinese Academy of Medical Sciences and Peking Union Medical College, Beijing 100050, China; 4 Department of Global Health, School of Public Health, Peking University, Beijing 100871, China

**Keywords:** voucher, screening, cardiovascular diseases, population, health policy

## Abstract

The present study aimed to evaluate the factors associated with unwillingness to join a healthcare voucher scheme for screening of cardiovascular risk factors in a Chinese population. We conducted a telephone survey by random selection of 1200 subjects who were aged 45 years or above in Hong Kong. We collected data on their attitude, perception, and perceived feasibility of a healthcare voucher scheme. The overall rates of having received at least one type, two types, and all three types of screening tests are 81.1%, 80.7%, and 79.3%, respectively. Younger individuals (aOR = 0.338, *p* = 0.004), those of a higher educational level (aOR = 1.825, *p* = 0.006), being employed (aOR = 3.030, *p* = 0.037), and lower perception of screening as beneficial (aOR = 0.495, *p* < 0.001) were significantly associated with no regular screening for at least one medical condition. The overall rate of willingness to join the voucher scheme (among those aged ≥ 45) is 83.7%. Male sex (aOR = 2.049, *p* = 0.010) and absence of family history of cardiovascular disease (aOR = 0.362, *p* = 0.002) are independent predictors of unwillingness to join. Our findings highlighted the significance of sex and family history on screening of cardiovascular factors. These constructs and independent predictors identified provide evidence-based formulation and implementation targeted screening strategies that enhance the screening rate of the three cardiovascular risk factors.

## 1. Introduction

Of the 40 million global deaths caused by non-communicable diseases (NCDs) in 2016, an estimated 32 million NCD-related deaths were attributable to diabetes, cardiovascular diseases, chronic respiratory diseases, and cancers [1]. Concurrently, a rising prevalence of multiple chronic conditions (MCC), shown to impose significant burdens on healthcare costs and utilisation, has been reported by epidemiological studies across the world [2]. Population ageing, improved diagnosis, and detection of diseases, changes to sedentary lifestyles, and increasing consumption of high-calorie diets are among the key contributors to this rising prevalence [3].

There are usually no signs or symptoms associated with high blood pressure, diabetes, or lipid disorders in the early stage, yet these disorders are recognised as important risk factors for cardiovascular diseases (CVD), stroke, and mortality [4]. Specifically, the lack of early detection contributes to late diagnosis of these chronic diseases, leading to the development of more severe complications [5]. To tackle these chronic conditions, early detection by regular screening has been proposed as an effective measure for earlier diagnosis of hypertension, diabetes, and hyperlipidaemia, which facilitates timely treatment and saves healthcare costs [6]. Screening is also essential for better long-term management, lowering avoidable hospital admissions, and alleviating the demand on healthcare utilization. All these favourable outcomes can eventually lead to more efficient health services.

The rising prevalence of hypertension, diabetes, and lipid disorders in younger age groups and the high proportion of undiagnosed individuals necessitate preventive care and early detection. The major onset of these three chronic diseases is commonly found at an age of 45 years [7], and the risk for people diagnosed with at least one chronic disease at this age is six times higher than that for younger people in Hong Kong [8]. Therefore, it is important for promote screening in this older population in Hong Kong. A recent simulation study in Thailand suggested that undiagnosed diabetes was most commonly found among those aged under 39 years, and that mortality of those with undiagnosed diabetes was ten-fold higher than that of those with diagnosed diabetes, supporting the importance of early detection and intervention [9]. Population statistics indicate the necessity for screening programmes to be targeted at ‘younger’ age groups to facilitate early detection and intervention, an implication consistent with study findings. Complementary studies to understand the key facilitators or barriers in health seeking behavior should also be considered among people of different age groups [10].

In Hong Kong, the increasing prevalence of NCDs poses significant challenges to our overburdened health system. Specifically, according to updated statistics from the Department of Health of the Hong Kong government, diseases associated with diabetes, lipid disorders, and heart diseases were among the top 10 leading causes of death in 2019 [11]. Notably, these three conditions are among the most commonly diagnosed chronic diseases in Hong Kong, and the co-occurrence of these diseases is widely observed. The 2014/15 Department of Health (DH) Population Health Survey showed an increase in prevalence of hypertension across age groups, even in a younger age group (35–44). Approximately 15.2% of individuals aged 35–44 were found to be hypertensive compared to the notable 26.7% among those aged 45–54. Importantly, as many as two thirds of all surveyed individuals had not previously been diagnosed as hypertensive [12]. Similar patterns have been observed for diabetes and lipid disorders. Diabetes continues to be a major cause of morbidity and mortality in Hong Kong, accounting for 0.9% of all deaths in 2017 [13]. The estimated prevalence of diabetes stands at approximately 10% of the local adult population [13] and 7.3% among those aged 45–54, of which 36.4% have not been previously diagnosed. A study by Quan et al. (2017) also demonstrated a rising prevalence of diabetes in Hong Kong, accounting for a significant rise in morbidity, premature mortality, and healthcare expenditure from 2006 to 2014 [14]. Additionally, the overall prevalence of pre-diabetes is increasing [15], a condition that could progress and lead to a diagnosis of diabetes if not detected and managed in a timely manner. Separately, based on the 2014 to 2015 Population Health Survey by the DH, an estimated 49.5% of the Hong Kong population aged 15 to 84 years had hyperlipidaemia, referring to elevated blood lipid levels and indicated by hypercholesterolemia. Yet, 70.2% of cases were undiagnosed before the administration of the health survey [7].

To address the widespread issues of undiagnosed chronic illness and the potential harm from absence of timely diagnosis internationally, screening for CVD-related chronic diseases have shifted to the forefront of governments’ efforts, resulting in adoption of screening programmes supported by clinical guidelines globally [16,17,18,19]. In the UK, people aged 40–74 receive complimentary health checks from the National Health Service (NHS), including measurement of blood pressure, glucose levels, plasma cholesterol, and BMI. Besides periodic health assessment, a nationwide NHS Diabetes Prevention Programme (NHS DPP) was also launched in 2016 to screen individuals over the age of 18 who are at high risk of developing Type 2 diabetes. This programme was a joint commitment from the NHS, Public Health England, and Diabetes UK. Although the programme is still undergoing, and the final evaluation report has not been released, one of its progress reports published recorded that over 40,000 people with non-diabetic hyperglycaemia conditions were referred to the programme for intervention [20]. In a separate example, an expert panel of diabetes expertise stakeholders recently proposed a new screening approach in Australia [21]. The Pharmacy Diabetes Screening Trial was established to provide community screening across regional remote areas and metropolitan of Australia, with thorough research to support the feasibility and value of pharmacy as a component of population screening efforts. Pharmacists would assess a patient’s risk of developing diabetes and refer the patient to their general practitioners for follow-up. The trial had successfully screened out more than 14,000 patients aged between 35 to 75 years and detected over 100 undiagnosed type 2 diabetes patients according to its preliminary results. Meanwhile, in the Asia Pacific region, the Ministry of Health in Singapore launched a national screening programme, named “Screen for Life (SFL)” in 2017. This SFL programme subsidizes citizens from age 25 and above to screen for hypertension, hyperlipidemia, diabetes, cervical cancer, and colorectal cancer, which helped screening for over 100,000 individuals with 40% identified as having potential chronic illness risk [22,23].

Rooting from the economic theories of supply and demand, the use of vouchers as a demand side mechanism increases utilisation of aims to health services for individuals with the need. Such mechanism enables the government to distribute resources in a more efficient manner by focusing on the targeted group, and it has been adopted as one of the effective means to promote health-seeking behaviour [24] This has been shown to be effective in overcoming financial, social and psychological barriers to facilitate the uptake of underutilised services prescribed by the scheme [25]. Additionally, the grounds were to encourage consumption of services, hence shifting the demand curve to the right and offering maximum positive externalities such as lowering long-term medical cost and increase in service quality to the society [26]. In other words, covering the majority of the populations and enrolling healthcare providers to see all voucher clients without ‘cherry picking’ will stimulate both supply and demand for need services which are the aim of the voucher programmes. Learning from international experiences, most voucher schemes implemented in low- or middle-income countries targeting specific health services (i.e., mental health services, reproductive health services) had been effective in promoting the utilisation and uptake rates of screening programmes [25,27,28,29,30,31]. Thus, building on the current healthcare voucher scheme, we envision the proposed population-based chronic disease screening voucher and disease management programme to be used by the Government as an instrument to not only promote early detection and disease management but also, importantly, to further promote well-coordinated public–private partnerships. This could shift the care burden from the public to private sector and thus alleviate the service demands of the overburdened public healthcare system [32].

The Elderly Health Care Voucher Scheme (EHCVS) in Hong Kong was launched as a pilot on 1 January 2009 and was converted into a standard recurrent programme in 2014 [33]. Currently, the scheme offers HKD 2000 to the resident population aged 65 and above every year, and each individual can accumulate unused entitlements up to HKD 8000. The scheme aims to incentivise the monetary incentives utilisation of primary care services in the private sector, allowing older people to seek healthcare from 10 categories of health professionals including western trained doctors, traditional Chinese medicine practitioners, nurse, physiotherapists, radiographers, optometrists, and dentists for both curative and preventive care [32,33]. The establishment of the EHCVS aims to enable private healthcare practitioners supplement the services in the public sector, thereby lowering the demand on the public sector [9,34]. However, over 90% of the voucher users reported using the voucher for acute care only, and less than 20% of the EHCVS participants used the vouchers for preventive care [32] when the voucher was first piloted. Studies have found a lack of confidence in the private healthcare services and the inadequacy of the healthcare voucher to cover all the consultation cost, which led to the current scheme, which has been enhanced and entitlements substantiality increased. Hence, the need to pay out-of-pocket expenses was a major reason for its low utilization level [32,35]. In light of this, building on the lessons learned from the EHCVS that is already in place, there is an opportunity for government to consider implementing a population-based chronic disease screening and management voucher programme. This not only promotes prevention through early detection of targeted chronic illnesses, but also supports follow-up chronic disease management plans that should take place in the community rather than in hospitals [36].

The current study aimed to evaluate the factors associated with not joining screening of cardiovascular risk factors and the unwillingness of joining a healthcare voucher scheme for promoting such screening in the Chinese population.

## 2. Materials and Methods

### 2.1. Subjects

All subjects who (1) were aged 45 years or above; (2) could communicate in either Cantonese, Putonghua, or English (which are the three most commonly used languages in Hong Kong); and (3) resided in a Hong Kong household at the time of the study were eligible to participate in this study.

### 2.2. Survey Instrument

The questionnaire consisted of survey items in Chinese (or Putonghua or English as applicable), which collected data on subjects’ attitude, perception, and perceived feasibility of the healthcare voucher scheme. Socio-demographic information, family and personal history of chronic diseases, self-perceived health status, and screening histories of chronic diseases were collected. The survey was designed based on qualitative interviews of relevant stakeholders in the proposed screening programme. Some of the constructs (attitudes, perceived benefits, and perceived health and psychological barriers) were developed based on the health belief model [37]. It was devised, pilot-tested and validated by a panel of epidemiologists, biostatisticians, physicians, and experts on healthcare systems.

The different parts of the validated survey include: (1) socio-demographic information: age, sex, educational level, marital status, employment status, and personal income; (2) family and personal history of chronic diseases, including hypertension, diabetes, and high blood cholesterol; (3) history of screening for hypertension, obesity, diabetes, and dyslipidaemia; (4) perceived benefits of chronic disease screening; and (5) perceived health or psychological barriers, including perceived lack of screening necessity, lack of screening benefits, busy working schedules, financial considerations, concerns about subsequent management of chronic diseases, and unawareness of screening venues.

### 2.3. Sampling Frame and Subject Recruitment

The sampling frame consists of all eligible subjects for the scheme. We purposively sampled an older population because of the nature of the study. Based on the sample size calculated, we used a computer randomizer to generate random numbers to select individuals for the surveys. The selection of the sample population was conducted by the Centre for Behavioural Research of the School of Public Health and Primary Care, The Chinese University of Hong Kong using simple random sampling.

One telephone number was considered one unit of randomization. Since a minority of family households had more than one fixed telephone line, all participants were asked if they have already been recruited in the survey to avoid double counting. Cell phone numbers were not included in the telephone directories for the present survey. If the target subject was not available, at least five call attempts in two different daytimes and three different evenings were given. If the target subject was available but too busy to receive the telephone interview, a mutually convenient time was scheduled to administer the survey.

According to standard methodology, only one subject was recruited from each household to avoid clustering effect within household [38]. Non-response was defined as non-completion of the survey after five telephone attempts. Non-respondents were replaced by the next subsequent household telephone number. The interviewed subjects were briefed about the purpose of the study, assured of the confidentiality of the telephone interview, and requested to provide informed consent. Enquiry telephone numbers of the survey were provided to the respondents for any inquiries arising from study participation.

### 2.4. Data Processing and Analysis

All the data were entered into a software spreadsheet and analysed by the Statistical Package for Social Sciences (SPSS) version 21.0. (IBM Corp, NY, US). A descriptive analysis of the socio-demographic data, clinical information, family history of chronic diseases, self-purchased health insurance, and the perceived adequacy of financial resource to pay healthcare expenditure was performed. The primary outcome variables included (1) not participating in screening for hypertension, diabetes, and lipid disorders; and (2) unwillingness to join the healthcare voucher scheme for hypertension, diabetes, and lipid screening. The association between each predictor variable was examined with the outcome variables by univariate analysis. Binary logistic regression models were constructed to examine the independent association between the predictors having *p* ≤ 0.10 in the univariate analysis and each outcome variable, separately. All *p* values ≤ 0.05 in the final regression model were regarded as statistically significant.

### 2.5. Sample Size Calculation

We assumed 50% as the proportion in all the outcomes which would provide the maximum sample size. A sample size of approximately 1200 participants could achieve a precision level of 0.03, from the formula: “precision = 1.96 × √[(p) × (1 − p)/N]”.

## 3. Results

### 3.1. Participant Characteristics

A total of 1200 respondents were recruited in the current study (Table 1). The majority of the individuals were aged ≥ 65 years (63.4%), followed by subjects aged 55–64 years (26.6%) and 45–54 years (10.0%). Most respondents were female (69.2%) and attained primary educational level or lower (45.3%). The majority were retired (54.0%) or were housewives (29.8%). Among them, 63.5% had been using medications for chronic diseases or were attending regular medical follow-up, where most of the healthcare consultations took place in the private sector (56.8%). Approximately 51.9% reported family history of hypertension, diabetes, lipid disorders, or stroke. The majority (74.5%) were not covered by self-purchased health insurance. A substantial proportion of the respondents perceived their financial resource to pay healthcare expenditure as inadequate (37.3%) and very inadequate (12.7%).

### 3.2. Factors Associated with Not Screening for Hypertension, Diabetes and Lipid Disorders

The overall rates of having received at least one, two, and all three types of screening test were 81.1%, 80.7%, and 79.3%, respectively. From univariate analysis (Table 2), younger age (cOR for 55–64 years: 0.550, 95% C.I. 0.355, 0.855, *p* = 0.008; cOR for ≥65 years: 0.191, 95% C.I. 0.125, 0.293, *p* < 0.001); higher educational level (cOR for secondary and tertiary educational level = 2.725 and 2.183, respectively); being employed (cOR for being employed = 2.033, 95% C.I. 1.399, 2.959, *p* < 0.001); non-recipients of comprehensive social security assistance (receipt of CSSA cOR 0.360, 95% C.I. 0.128, 1.099, *p* = 0.052); perception of screening being beneficial (cOR 0.565, 95% C.I. 0.411, 0.778, *p* < 0.001); and family history without chronic diseases (cOR 0.422, 95% C.I. 0.310, 0.573, *p* < 0.001; referent: family history with chronic diseases) were significant covariates. In the multiple regression analysis, younger individuals (aOR 0.338, 95% C.I. 0.161, 0.711, *p* = 0.004; referent: subjects aged ≥65 years); higher educational level (aOR for secondary and tertiary educational level = 1.825 and 1.391, respectively); being employed (aOR = 3.030, 95% C.I. 1.068, 8.621, *p* = 0.037); lower perception of screening as beneficial (aOR 0.495, 95% C.I. 0.345, 0.710, *p* < 0.001); older individuals who have a family history of chronic diseases (aOR for ≥65 years by with family history = 0.284, 95% C.I. 0.109, 0.736, *p* = 0.010); and employed individuals with high educational level (aOR for secondary by employed = 0.270, 95% C.I. 0.084, 0.864, *p* = 0.027; aOR for tertiary by employed = 0.136, 95% C.I. 0.034, 0.548, *p* = 0.005) were significantly associated with no regular screening for at least one medical condition. The significant covariates from regression analysis for not having received at least two (Table 3) or all three types (Table 4) of screening tests were similar.

### 3.3. Factors Associated with Unwillingness to Join the Voucher Scheme Programme for Screening

The overall rates of willingness to join the voucher scheme (among those aged ≥ 45) is 83.7%. We excluded subjects who were attending regular follow-up in clinics for hypertension, diabetes, lipid disorders, or stroke. In univariate analysis of absence of willingness to join the EHCVS for preventive screening (Table 5), male individuals (cOR = 2.198, 95% C.I. 1.309, 3.690, *p* = 0.003) and those without family history of the above-mentioned chronic diseases (cOR = 0.341, 95% C.I. 0.182, 0.638, *p* = 0.001; referent: with family history) were significantly more likely to express unwillingness to join the screening programme. Being male (aOR = 2.049, 95% C.I. 1.183, 3.546, *p* = 0.010) and the absence of family history (aOR = 0.362, 95% C.I. 0.192, 0.680, *p* = 0.002; referent: with family history) remained to be the significant predictors of unwillingness to receive screening for conditions in the multivariate regression model (Table 5).

To further analyse the results by gender (Table 6), we found fewer males were willing to participate in the voucher scheme compared with females (75.6% vs. 87.2%, *p* = 0.003). Male subjects were more likely to be employed (42.7% vs. 15.2%, *p* < 0.001), of high income (34.2% vs. 17.5%, *p* < 0.001), without family history of cardiovascular diseases (67.2% vs. 57.3%, *p* = 0.039), and with health insurance (48.4% vs. 37.6%, *p* = 0.039).

## 4. Discussion

From this telephone survey, we found that the overall rates of having received at least one type, two types, and all three types of screening test were 81.1%, 80.7%, and 79.3%, respectively, among those aged ≥ 45. Consistent with this finding, the overall rate of willingness to join the voucher scheme for screening was 83.7%. Several independent factors, including age, education level, employment status, social security assistance, and perception of the benefits of the screening and family history, were associated with not having received one, two, and all three types of screening test. With interaction effect being considered, younger individuals who did not have a family history of any of the three chronic diseases was associated with not having received screening for at least one medical condition. We also identified that independent factors of males and individuals who did not have family history of any of the three chronic diseases were associated with unwillingness to join a proposed screening programme. Since chronic disease is best managed at a younger age, it is important for us to identify the at-risk population and promote screening among them. However, the perceived risk of chronic diseases was usually lower in the young population. Therefore, the use of healthcare voucher and community education to increase awareness could be useful for promoting screening programmes of chronic diseases.

Counterintuitively, in the multivariate analysis with variable interactions considered, we also found that employed persons and those with secondary education and above were also more likely not to have regular screening for the three medical conditions. One possible explanation could be they were busy and experienced time constraints in identifying and accessing structured screening programmes designed from established guidelines and perceived as benefit. Further studies may be needed to confirm this concomitantly. Consideration should be given by policy makers in providing better health promotion information on the recommended screening programmes of how and where these could be accessed.

This is the one of the few representative population-based studies in Hong Kong that examined the factors affecting participant’s willingness to participate in hypertension, diabetes, and lipid disorder screening. It has a high response rate based on a random sampling strategy, and the survey instrument was validated by an expert panel. There are, nevertheless, several limitations that should be addressed. Firstly, this is a cross-sectional survey, and we could not establish a cause-and-effect relationship between the independent factors and the outcome. They could, however, be used in prediction of screening intention. Additionally, this survey examined risk associations in a Chinese population, and the generalizability of its findings to other settings should be approached cautiously. Finally, since the telephone survey relies on landline and there is a possibility of selection bias, although more than 91% of all Hong Kong residents have access to landlines [31].

Based on this study, it was found that male subjects without family history of chronic disease were less likely to receive hypertension, diabetes, and lipid disorder screening as compared to other individual factors. From this study, we found that males were less likely to join the voucher scheme in the future. Possible explanations include they were more likely to be employed and thus may have had limited time for the scheme for screening. Additionally, they tend to have higher income and health insurance, which may reduce the incentive effects of healthcare voucher. In addition, they have a lower perceived risk of cardiovascular diseases, as fewer male subjects had such a family history when compared with the females. Policymakers should allocate resources or raising awareness of the importance of screening for these disorders and provide health information on the recommended screening programmes and how and when they could be accessed by younger adults and men as well as those without any family history through promotional campaigns and health education. While it was observed that family history is associated with an increase in incidence of hypertension, diabetes, and lipid disorder, the overall incidence rate has also increased over the past decades with a shift towards younger patients, suggesting the need for early detection [39,40,41,42]. Having three types of screening is essential to detect and treat early and to identify potential high-risk individuals and provide proper recommendation for lowering their risk. This will not only benefit the patients but also benefit population health, lowering the demand on the healthcare system in the future and reducing the operation cost associated with these chronic diseases.

The results of the current study may inform how to use EHVCS to promote screening for cardiovascular risk factors among the older population in Hong Kong. However, the EHVCS alone may not be enough for promoting screening. Other strategies, including organised educational programmes and interactive reminders from physicians, could be useful for improving regular screening participation in the targeted group. It is recommended to disseminate more educational information regarding screening for cardiovascular designed to increase health awareness and reduce relevant barriers to promote their intention to join screening. To ensure a more extensive coverage and equity of access, organised educational programmes may be preferred, including health talks and seminars, newspaper advertisements, leaflets, or focus group interviews. A systematic reach of the high-risk population by invitations from general practice doctors could also help to promote the screening. For non-participants of screening programmes, sending additional reminders by healthcare professionals may be particularly effective. Interactive telephone reminders are believed to be more effective compared with short message services, WhatsApp, or emails. The above strategies could focus on younger individuals, subjects with higher educational levels, and those employed, as they were less likely to join in the screening for cardiovascular risk factors according to the current study.

## 5. Conclusions

In summary, this study has examined the factors independently associated with no participation in screening for hypertension, diabetes, and lipid disorders and their unwillingness to join the voucher scheme for screening. Our findings highlighted the significance of gender and family history on participation and acceptance of screening. The constructs and independent predictors identified provide evidence-based formulation and implementation of targeted screening strategies that enhance screening uptake and thus lower the impact of hypertension, diabetes, and lipid disorder on the health and demands on the healthcare system in the future.

## Figures and Tables

**Table 1:** Characteristics of the respondents.

	n	%
**Age (years)**		
45–54	120	10.0
55–64	319	26.6
≥65	761	63.4
**Gender**		
Male	370	30.8
Female	830	69.2
**Educational level**		
Primary or below	543	45.3
Secondary	479	39.9
Tertiary or above	154	12.8
Refused to answer	24	2
**Job status**		
Full-time or part-time	164	13.7
Retired	648	54.0
Housewife	358	29.8
Student	0	0
Unemployed	22	1.8
Refused to answer	8	0.7
**Monthly personal income (HKD)**		
<10,000	20	1.7
10,000–19,999	47	3.9
20,000–29,000	33	2.8
30,000–60,000	22	1.8
>60,000	10	0.8
Unstable income	6	0.5
Refused to answer	26	2.2
N/A as no current job	1036	86.3
**Recipient of CSSA**		
Yes	52	4.3
No	1140	95.0
Refused to answer	8	0.7
**Regular follow-up or use of medication for chronic diseases**		
Yes	762	63.5
No	436	36.3
Refused to answer	2	0.2
**Healthcare consultations mainly in**		
Public sector	274	22.8
Private sector	682	56.8
Public or private (more or less equal)	194	16.2
Don’t know/no opinions	38	3.2
Others (Chinese Medicine, over-the-counter drugs)	12	1.0
**Family history of hypertension, diabetes, lipid disorders, or stroke**		
Yes	623	51.9
No	492	41.0
Don’t know/no opinions	78	6.5
Refused to answer	7	0.6
**Medical insurance provided by employers**		
Yes	87	7.2
No	70	5.8
Not applicable (no employers)	1031	85.9
Don’t know/no opinions	1	0.1
Refused to answer	11	0.9
**Self-purchased health insurance**		
Yes, Voluntary Health Insurance Scheme (VHIS)	3	0.3
Yes, personal health insurance	253	21.1
Both VHIS and personal health insurance	9	0.8
No	894	74.5
Don’t know/no opinions	0	0.0
Refused to answer	41	3.4
**Perceived adequacy of financial resource to pay healthcare expenditure**		
More than adequate	9	0.8
Adequate	166	13.8
Just enough	196	16.3
Inadequate	447	37.3
Very inadequate	152	12.7
Don’t know/no opinions	212	17.7
Refused to answer	18	1.5

**Table 2:** Factors associated with not screening for at least one factor.

**Univariate Analysis**		**n**	**%**	**COR**	**95%** C.I.		**Sig.**
**Age**	45–54	120	40.8	Reference			
	55–64	316	27.5	0.550	0.355	0.855	0.008
	65 or above	747	11.6	0.191	0.125	0.293	<0.001
**Sex**	Male	365	19.2	1.031	0.753	1.412	0.847
	Female	818	18.7	Reference			
**Educational Level**	Primary or below	531	11.5	Reference			
	Secondary	475	26.1	2.725	1.946	3.817	<0.001
	Tertiary or above	154	22.1	2.183	1.372	3.472	0.001
**Job Status**	Employed	164	29.3	2.033	1.399	2.959	<0.001
	Not employed (including unemployed, homemaker, retired)	1011	16.9	Reference			
**Income**	Below 20,000	67	31.3	Reference			
	20,000–30,000	33	30.3	0.952	0.386	2.353	0.916
	Above 30,000	32	15.6	0.406	0.137	1.200	0.103
**CSSA**	Yes	51	7.8	0.360	0.128	1.009	0.052
	No	1124	19.1	Reference			
**Perceive screening as beneficial**		1169	18.6	0.565	0.411	0.778	<0.001
**Family history of hypertension, diabetes, lipid disorders, or stroke**	With family history	616	13.3	0.422	0.310	0.573	<0.001
	Without family history	487	26.7	Reference			
**Insurance**	With insurance	294	19.7	2.299	0.816	1.603	0.435
	Without insurance	848	17.7	Reference			
**Multivariate Analysis**		**n**	**%**	**AOR**	**95% C.I.**		**Sig.**
**Age**	45–54	111	38.7	Reference			
	55–64	298	27.9	0.749	0.366	1.534	0.429
	65 or above	658	11.6	0.338	0.161	0.711	0.004
**Educational Level**	Primary or below	468	11.5	Reference			
	Secondary	454	25.8	1.825	1.189	2.801	0.006
	Tertiary	145	21.4	1.391	0.750	2.584	0.295
**Job Status**	Employed	157	28.7	3.030	1.068	8.621	0.037
	Not employed (including unemployed, homemaker, retired)	910	17.3	Reference			
**Perceive screening as beneficial**		1067	18.9	0.495	0.345	0.710	<0.001
**Family history of hypertension, diabetes, lipid disorders or stroke**	With family history	608	13.3	0.962	0.436	2.128	0.925
	Without family history	459	26.4	Reference			
**Age × Family History**	55–64 by with family history	171	21.1	0.476	0.184	1.233	0.127
	65 or above by with family history	379	5.8	0.284	0.109	0.736	0.010
**Education level × Job Status**	Secondary by employed	89	33.7	0.270	0.084	0.864	0.027
	Tertiary by employed	47	21.3	0.136	0.034	0.548	0.005

**Table 3:** Factors associated with not screening for at least two factors.

**Univariate Analysis**		**n**	**%**	**COR**	**95% C.I.**		**Sig.**
**Age**	45–54	120	40.8	Reference			
	55–64	316	28.5	0.577	0.372	0.894	0.014
	65 or above	747	11.9	0.196	0.128	0.300	<0.001
**Sex**	Male	365	19.5	1.016	0.744	1.389	0.917
	Female	818	19.2	Reference			
**Educational Level**	Primary or below	531	11.9	Reference			
	Secondary	475	26.7	2.710	1.946	3.774	<0.001
	Tertiary or above	154	22.1	2.105	1.325	3.344	0.002
**Job Status**	Employed	164	29.3	2.037	1.403	2.950	<0.001
	Not employed (including unemployed, homemaker, retired)	1011	16.9	Reference			
**Income**	Below 20,000	67	32.8	Reference			
	20,000–30,000	33	30.3	0.890	0.361	2.188	0.799
	Above 30,000	32	15.6	0.379	0.128	1.117	0.079
**CSSA**	Yes	51	7.8	0.350	0.125	0.981	0.046
	No	1124	19.6	Reference			
**Perceive screening as beneficial**		1169	19	0.579	0.423	0.793	0.001
**Family history of hypertension, diabetes, lipid disorders, or stroke**	With family history	616	14	0.441	0.326	0.597	<0.001
	Without family history	487	26.9	Reference			
**Insurance**	With insurance	294	20.1	1.131	0.810	1.580	0.469
	Without insurance	848	18.2	Reference			
**Multivariate Analysis**		**n**	**%**	**AOR**	**95% C.I.**		**Sig.**
**Age**	45–54	111	38.7	Reference			
	55–64	298	28.9	0.739	0.361	1.511	0.407
	65 or above	658	11.9	0.344	0.164	0.721	0.005
**Educational Level**	Primary or below	468	12	Reference			
	Secondary	454	26.4	1.873	1.227	2.865	0.004
	Tertiary	145	21.4	1.353	0.732	2.506	0.334
**Job Status**	Employed	157	29.3	3.597	1.304	9.901	0.013
	Not employed (including unemployed, homemaker, retired)	910	17.7	Reference			
**Perceive screening as beneficial**		1067	19.4	0.507	0.355	0.724	<0.001
**Family history of hypertension, diabetes, lipid disorders, or stroke**	With family history	608	14	0.964	0.437	2.128	0.928
	Without family history	459	26.6	Reference			
**Age × Family History**	55–64 by with family history	171	22.8	0.525	0.204	1.355	0.183
	65 or above by with family history	379	6.1	0.288	0.112	0.745	0.010
**Education level × Job Status**	Secondary by employed	89	32.6	0.217	0.070	0.677	0.008
	Tertiary by employed	47	19.1	0.117	0.030	0.461	0.002

**Table 4:** Factors associated with not screening for all three factors.

**Univariate Analysis**		**n**	**%**	**COR**	**95% C.I.**		**Sig.**
**Age**	45–54	120	43.3	Reference			
	55–64	316	30.4	0.571	0.370	0.880	0.011
	65 or above	747	13	0.195	0.128	0.297	<0.001
**Sex**	Male	365	20.8	1.010	0.745	1.368	0.949
	Female	818	20.7	Reference			
**Educational Level**	Primary or below	531	13.7	Reference			
	Secondary	475	27.6	2.387	1.736	3.289	<0.001
	Tertiary or above	154	24	1.984	1.272	3.096	0.003
**Job Status**	Employed	164	31.7	2.033	1.410	2.924	<0.001
	Not employed (including unemployed, homemaker, retired)	1011	18.6	Reference			
**Income**	Below 20,000	67	34.3	Reference			
	20,000–30,000	33	30.3	0.832	0.339	2.041	0.687
	Above 30,000	32	18.7	0.442	0.159	1.225	0.116
**CSSA**	Yes	51	9.8	0.411	0.162	1.046	0.062
	No	1124	20.9	Reference			
**Perceive screening as beneficial**		1169	20.4	0.611	0.451	0.827	0.001
**Family history of hypertension, diabetes, lipid disorders, or stroke**	With family history	616	15.1	0.450	0.335	0.605	<0.001
	Without family history	487	28.3	Reference			
**With insurance or not**	With insurance	294	21.8	1.143	0.826	1.582	0.419
	Without insurance	848	19.6	Reference			
**Multivariate Analysis**		**n**	**%**	**AOR**	**95% C.I.**		**Sig.**
**Age**	45–54	111	41.4	Reference			
	55–64	298	30.5	0.829	0.407	1.686	0.605
	65 or above	658	12.6	0.345	0.165	0.719	0.004
**Educational Level**	Primary or below	468	13.5	Reference			
	Secondary	454	27.1	1.634	1.080	2.469	0.020
	Tertiary	145	23.4	1.297	0.713	2.358	0.394
**Job Status**	Employed	157	31.2	3.381	1.412	10.417	0.008
	Not employed (including unemployed, homemaker, retired)	910	18.8	Reference			
**Perceive screening as beneficial**		1067	20.6	0.513	0.362	0.728	<0.001
**Family history of hypertension, diabetes, lipid disorders or stroke**	With family history	608	15	1.212	0.553	2.653	0.631
	Without family history	459	28.1	Reference			
**Age × Family History**	55–64 by with family history	171	23.4	0.370	0.145	0.945	0.038
	65 or above by with family history	379	6.6	0.236	0.093	0.600	0.002
**Education level × Job Status**	Secondary and employed	89	33.7	0.200	0.065	0.615	0.005
	Tertiary and employed	47	21.3	0.106	0.028	0.404	0.001

**Table 5:** Factors associated with unwillingness participate in voucher scheme for screening.

**Univariate Analysis**		**n**	**%**	**COR**	**95% C.I.**		**Sig.**
**Age**	45–54	92	15.2	Reference			
	55–64	178	14	0.911	0.448	1.848	0.795
	65 or above	173	19.1	1.314	0.663	2.604	0.435
**Sex**	Male	131	24.4	2.198	1.309	3.690	0.003
	Female	312	12.8	Reference			
**Educational Level**	Primary or below	104	14.4	Reference			
	Secondary	241	17.4	1.252	0.660	2.375	0.491
	Tertiary or above	87	13.8	0.950	0.419	2.155	0.901
**Job Status**	Employed	100	20	1.370	0.773	2.427	0.730
	Not employed (including unemployed, homemaker, retired)	337	15.4	Reference			
**Income**	Below 20,000	37	18.9	Reference			
	20,000–30,000	21	23.8	1.339	0.366	4.902	0.659
	Above 30,000	20	20	1.072	0.272	4.219	0.921
**CSSA**	Yes	4	25	1.739	0.178	16.949	0.634
	No	435	16.1	Reference			
**Perceive screening as beneficial**		437	16	0.900	0.530	1.527	0.696
**Family history of hypertension, diabetes, lipid disorders or stroke**	With family history	171	8.2	0.341	0.182	0.638	0.001
	Without family history	251	20.7	Reference			
**Insurance**	With insurance	172	18	1.266	0.751	2.132	0.377
	Without insurance	250	14.8	Reference			
**Multivariate Analysis**		**n**	**%**	**AOR**	**95% C.I.**		**Sig.**
**Gender**	Male	122	23.8	2.049	1.183	3.546	0.010
	Female	300	12.3	Reference			
**Family history of hypertension, diabetes, lipid disorders or stroke**	With family history	171	8.2	0.362	0.192	0.680	0.002
	Without family history	251	20.7	Reference			

**Table 6:** Sex-specific willingness of participation in voucher scheme for screening.

		Male		Female			
		n	%	n	%	χ^2^	*p*
**W** **illingness to participate**	Unwilling	32	24.4	40	12.8		
	Willing	99	75.6	272	87.2	9.13	0.003
**Age**	45–54	32	24.4	60	19.2		
	55–64	48	36.6	130	41.7		
	65 or above	51	38.9	122	39.1	1.78	0.411
**Educational Level**	Primary or below	23	18.1	81	26.6		
	Secondary	71	55.9	170	55.7		
	Tertiary or above	33	26.0	54	17.7	5.71	0.058
**Job Status**	Employed	41	42.7	47	15.2		
	Not employed (including unemployed, homemaker, retired)	55	57.3	262	84.8	32.56	<0.001
**Income**	Below 20,000	9	23.7	28	70.0		
	20,000–30,000	16	42.1	5	12.5		
	Above 30,000	13	34.2	7	17.5	17.28	<0.001
**CSSA**	Yes	0	0.0	4	1.3		
	No	128	100.0	307	98.7	na	0.327
**Family history of hypertension, diabetes, lipid disorders or stroke**	With family history	40	32.8	131	43.7		
	Without family history	82	67.2	169	56.3	4.26	0.039
**Insurance**	With insurance	60	48.4	112	37.6	4.26	0.039
	Without insurance	64	51.6	186	62.4		

## Data Availability

The data are available upon reasonable request from the corresponding authors.
